# Bi-specific splice-switching PMO oligonucleotides conjugated via a single peptide active in a mouse model of Duchenne muscular dystrophy

**DOI:** 10.1093/nar/gku1256

**Published:** 2014-12-02

**Authors:** Fazel Shabanpoor, Graham McClorey, Amer F. Saleh, Peter Järver, Matthew J.A. Wood, Michael J. Gait

**Affiliations:** 1 Medical Research Council, Laboratory of Molecular Biology, Francis Crick Avenue, Cambridge CB2 0QH, UK; 2 Department of Physiology, Anatomy and Genetics, University of Oxford, South Parks Road, Oxford OX1 3QX, UK

## Abstract

The potential for therapeutic application of splice-switching oligonucleotides (SSOs) to modulate pre-mRNA splicing is increasingly evident in a number of diseases. However, the primary drawback of this approach is poor cell and *in vivo* oligonucleotide uptake efficacy. Biological activities can be significantly enhanced through the use of synthetically conjugated cationic cell penetrating peptides (CPPs). Studies to date have focused on the delivery of a single SSO conjugated to a CPP, but here we describe the conjugation of two phosphorodiamidate morpholino oligonucleotide (PMO) SSOs to a single CPP for simultaneous delivery and pre-mRNA targeting of two separate genes, exon 23 of the *Dmd* gene and exon 5 of the *Acvr2b* gene, in a mouse model of Duchenne muscular dystrophy. Conjugations of PMOs to a single CPP were carried out through an amide bond in one case and through a triazole linkage (‘click chemistry’) in the other. The most active bi-specific CPP–PMOs demonstrated comparable exon skipping levels for both pre-mRNA targets when compared to individual CPP–PMO conjugates both in cell culture and *in vivo* in the *mdx* mouse model. Thus, two SSOs with different target sequences conjugated to a single CPP are biologically effective and potentially suitable for future therapeutic exploitation.

## INTRODUCTION

Splice-switching oligonucleotides (SSOs) are currently very promising for therapeutic use in both Duchenne muscular dystrophy (DMD) through exon skipping and for Spinal muscular atrophy (SMA) through promotion of exon inclusion. Such SSOs are designed to sterically block splice sites or specific binding motifs for splicing machinery in order to promote exon inclusion or exclusion. For DMD, targeting of the dystrophin pre-mRNA with a SSO is used to ‘skip’ an exon that contains a nonsense coding mutation, or to remove an exon neighbouring an out-of-frame genomic deletion, so as to restore the mRNA reading frame. This allows synthesis of an internally deleted dystrophin protein that retains the elements crucial for function (1–3).

Proof-of-principle of this approach has been demonstrated in murine and canine animal models of DMD (4–7) and more recently in human phase II/III clinical trials (8–12). Various SSO chemistries have been developed for exon skipping including phosphorodiamidate morpholino oligonucleotides (PMO), peptide nucleic acids (PNA), locked nucleic acid (LNA), 2′-*O*-methoxyethyl phosphorothioate oligonucleotides (2′-MOE/PS), 2′-*O*-methyl phosphorothioate oligonucleotides (2′-OMe/PS) and tricyclo oligonucleotides (see (2) for a recent review). The two major SSO chemistries that have been used more extensively and which are in current clinical trials in DMD patients are 2′-OMe/PS (9,11) and PMO (8,10,12). In these trials, production of dystrophin protein has been demonstrated, albeit at low levels, and rather disappointingly a recent phase III trial with 2′-OMe-PS SSO was not able to meet its primary endpoint of a statistically significant improvement in the 6 min walk test (13). Nevertheless, preliminary results with PMO chemistry are promising and further studies are planned to target additional exons (8,9). To date, clinical trials have focused on targeted removal of exon 51, since this would benefit the largest patient pool, but SSOs are also being developed to target other exons between 45 and 55.

The most significant barrier to success for splice-switching therapies has been effective delivery. In the case of PMO, and indeed other chemistries, the level of exon skipping, and hence the amount of functional dystrophin restored in muscle, is poor unless high doses are used. This is thought to be due mostly to their rapid clearance from the body following systemic administration, as well as their poor ability to penetrate cellular barriers and reach their nuclear target site. One approach to address this has been to attach cell-penetrating peptides (CPPs) that can effectively carry charge neutral PMO cargos across cell membranes to their pre-mRNA target site in the nucleus. In particular, PMOs conjugated to arginine-rich CPPs (known as P-PMOs) have been shown to enhance dystrophin production in muscle following systemic administration in the *mdx* mouse model of DMD (14–16). We have previously developed a series of novel arginine-rich CPPs known as PNA/PMO internalization peptides (Pips), comprised of two arginine-rich sequences separated by a central short hydrophobic core sequence. These Pip peptides were designed to improve serum stability whilst maintaining a high level of exon skipping, initially by attachment to a PNA cargo (17). Further derivations of these peptides were designed as conjugates of PMOs, which were shown to lead to high body-wide skeletal muscle dystrophin production, and importantly also including the heart, following systemic administration (5). A later version (Pip6a-PMO) proved to be an even more efficient conjugate in mediating dystrophin production in the *mdx* mouse (18).

Whilst restoration of the absent dystrophin protein is the primary goal for genetic therapies for DMD, consideration of complementary therapies to reduce pathological features of disease or to improve muscle function are also very important. One such strategy would be to promote muscle growth through targeting of the myostatin pathway. Myostatin, or growth/differentiation factor-8 (GDF8), is a member of the transforming growth factor-β (TGF-β) family, which is involved in muscle homeostasis and acts to inhibit muscle growth (19). Myostatin is involved in control of myogenesis through binding to activin type IIB receptor (AcvRIIb) (20,21) which recruits and activates activin type I receptor (ALK4 or ALK5) (22). Activin receptor activation results in phosphorylation of intracellular signalling mediators Smad2 and Smad3 that translocate to the nucleus to affect gene transcription. There is evidence that inhibition of the myostatin pathway has the potential for clinical benefit in DMD. Transgenic knockout models for both myostatin and dystrophin demonstrate increased musculature due to fibre hypertrophy as well as reduced fibrosis and fat deposition, compared to *mdx* mice alone (23). Similarly, when *mdx* mice were treated with anti-myostatin antibodies this resulted in enlarged muscles concurrent with improved muscle function and strong reduction in diaphragm fibrosis (24). Based on these improved muscle features, the concept of myostatin down-regulation concurrent with dystrophin restoration has been investigated. Dumonceaux *et al.* reported the use of Adeno-associated virus (AAV) constructs to combine RNAi-mediated down-regulation of AcvRIIB with a U7-based small RNA exon skipping technique to restore dystrophin (25). Whilst concurrent treatment did not improve muscle mass, absolute and specific forces were much greater compared to either individual strategy. A subsequent study by Hoogaars *et al.*, utilized soluble AcvRIIB decoy receptors in combination with AAV-U7 mediated dystrophin restoration to treat *mdx* mice (26). Treatment with decoy AcvRIIB increased body weight, with morphometric measurements of muscle fibres suggesting that muscle growth was due to hypertrophy (20,21,27). Based on the potential success of this approach, we sought to simultaneously target both the dystrophin and the myostatin pathway as a molecular model to evaluate the efficacy of using bi-specific PMO compounds.

A splice-switching approach has been used previously to target and down-regulate expression in the myostatin pathway, with SSOs developed to target both the *Mstn* (28) and *Alk5* (29) transcripts. Whilst the principle of multiple-exon targeting for both dystrophin restoration and myostatin depletion has been demonstrated before using a cocktail of individual SSOs (30–32), there are advantages in development of a bi-specific compound to target two pre-mRNAs. First, the outcome of use of a bi-specific SSO is that both SSOs must enter the same cell, whereas for a cocktail of SSOs there will likely be a mixed population of cells where either none, one or both genes are targeted. More importantly, since cell and *in vivo* toxicity of P-PMOs is thought to be predominantly due to that of the peptide, the use of a single peptide to deliver both PMOs halves the total peptide requirement compared to use of a cocktail of two separate P-PMOs, and this may help to reduce the potential for peptide-mediated toxicity. Note also that two 2′-OMe/PS SSOs that target both dystrophin and myostatin have been joined together recently to make a bi-specific construct, but without any attached delivery peptide, but in this case activity in *mdx* mice was not seen for the dystrophin SSO (33).

In our study, we selected Pip6a (18) as the CPP to simultaneously deliver two different PMOs as a bi-specific conjugate and to develop the chemistries for their attachment. Three different conjugation chemistries including amide, disulfide and triazole bonds were utilized to allow orthogonal conjugation. The first PMO targets exon 23 of the dystrophin gene to correct the *mdx* genotype and the second to target removal of exon 5 of the *Acvr2b* gene so as to produce an internally deleted protein that lacks the crucial trans-membrane domains. Several different bi-specific conjugate designs were investigated whereby two PMOs were joined either at one end of the Pip6a peptide or with one PMO at either N- or C-termini. The activities of these conjugate constructs were assessed in mouse *mdx* cells and the most active bi-specific conjugates (D2 and D3), which had both PMOs attached at the C-terminus of the CPP, were shown to have closely comparable *Dmd* exon skipping activity to the single Pip6a-PMO targeting *Dmd*. D2 and D3 conjugates were also assessed in the same cells for targeting of *Acvr2b* and both conjugates demonstrated only very slightly reduced exon skipping activity compared to Pip6a-PMO targeting *Acvr2b*. Importantly, the cell viability using a bi-specific compound was significantly better than for a mixture of the two individual Pip6a-PMOs. We furthermore assessed the potential of this approach in an *in vivo* environment through intramuscular administration and demonstrated that there were no significant differences in exon skipping activities for both *Dmd* and *Acvr2b* targets between bi-specific conjugates (D2 and D3) and a cocktail of the individual P-PMO equivalents.

## MATERIALS AND METHODS

### Materials

Fmoc-protected amino acids, coupling reagents (HBTU and PyBOP) and the Fmoc-β-Ala-OH preloaded Wang resin (0.19 mmol g^−1^) were obtained from Merck (Hohenbrunn, Germany). Fmoc-azido-l-lysine-OH was from IRIS Biotech GMBH (Deutschland, Germany). Fmoc-l-bis-homopropargylglycine-OH (Bpg) was purchased from Chiralix (Nijmegen, The Netherlands). Chicken embryo extract (CEE) and horse serum (HS) for cell culture were obtained from Sera Laboratories International Ltd (West Sussex, UK). γ-Interferon was obtained from Roche Applied Science (Penzberg, Germany). All other reagents were obtained from Sigma–Aldrich (St Louis, MO, USA) unless otherwise stated. MALDI-TOF mass spectrometry (Table 1) was carried out using a Voyager DE Pro BioSpectrometry workstation. A stock solution of 10 mg ml^−1^ of α-cyano-4-hydroxycinnamic acid or sinapinic acid in 60% acetonitrile in water was used as matrix. The measurements have an accuracy level of ±0.1%.

**Table 1.** Calculated and experimentally found values for masses of peptide derivatives, functionalized PMOs and conjugates

	[M+H]^+^	
	Calculated	Experimental
Peptides		
Bpg-Pip6a	3074.0	3075
Pip6a-X-Bpg-B	3253.5	3254
Pip6a-X-C-B	3239.7	3241
Functionalized PMO		
3′-Azido-PMO (*Acvr2b*)	8575.4	8575
3′-Azido-PMO *(Dmd*)	8567.4	8567
3′-Npys-PMO *(Dmd)*	8663.1	8664
Peptide-PMO		
Bpg-Pip6a-PMO (*Dmd*)	11470.8	11471
Pip6a-X-Bpg-B-PMO *(Dmd)*	11648.5	11649
Pip6a-X-Bpg-B-PMO *(Acvr2b)*	11655.0	11656
Pip6a-X-C-B-PMO *(Acvr2b)*	11641.7	11642
Bi-specific conjugates		
D1	20046.2	20048
D2	20223.9	20225
D3	20222.4	20224
D4	20150.8	20151

**Figure 1.**
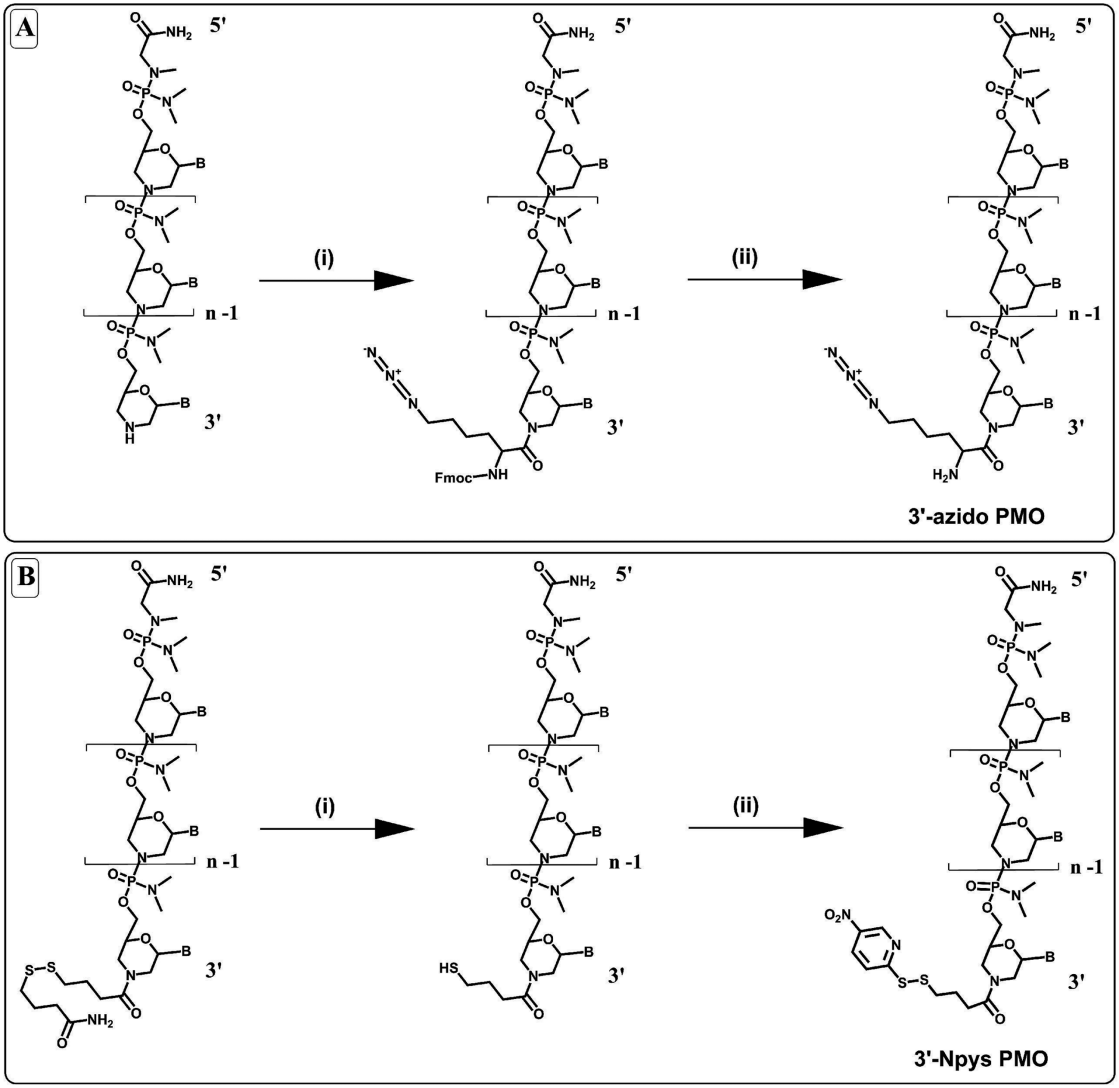
Functionalization of the second PMO: (**A**) (i) conjugation of Fmoc-azido-l-lysine-OH to the 3′-end of unmodified PMO and (ii) removal of the Fmoc group using 20% piperidine. (**B**) (i) Reduction of the disulphide bond of 3′-disulphide functionalized PMO by treatment with a 10-fold excess of TCEP and (ii) activation of the resultant free thiol group using a 2.5-fold molar excess of 2,2′-dithiobis(5-nitropyridine) (DTNP) in DMSO: acetonitrile (0.1% TFA):H_2_O (0.1% TFA) with (1:1:3) ratios.

**Figure 2.**
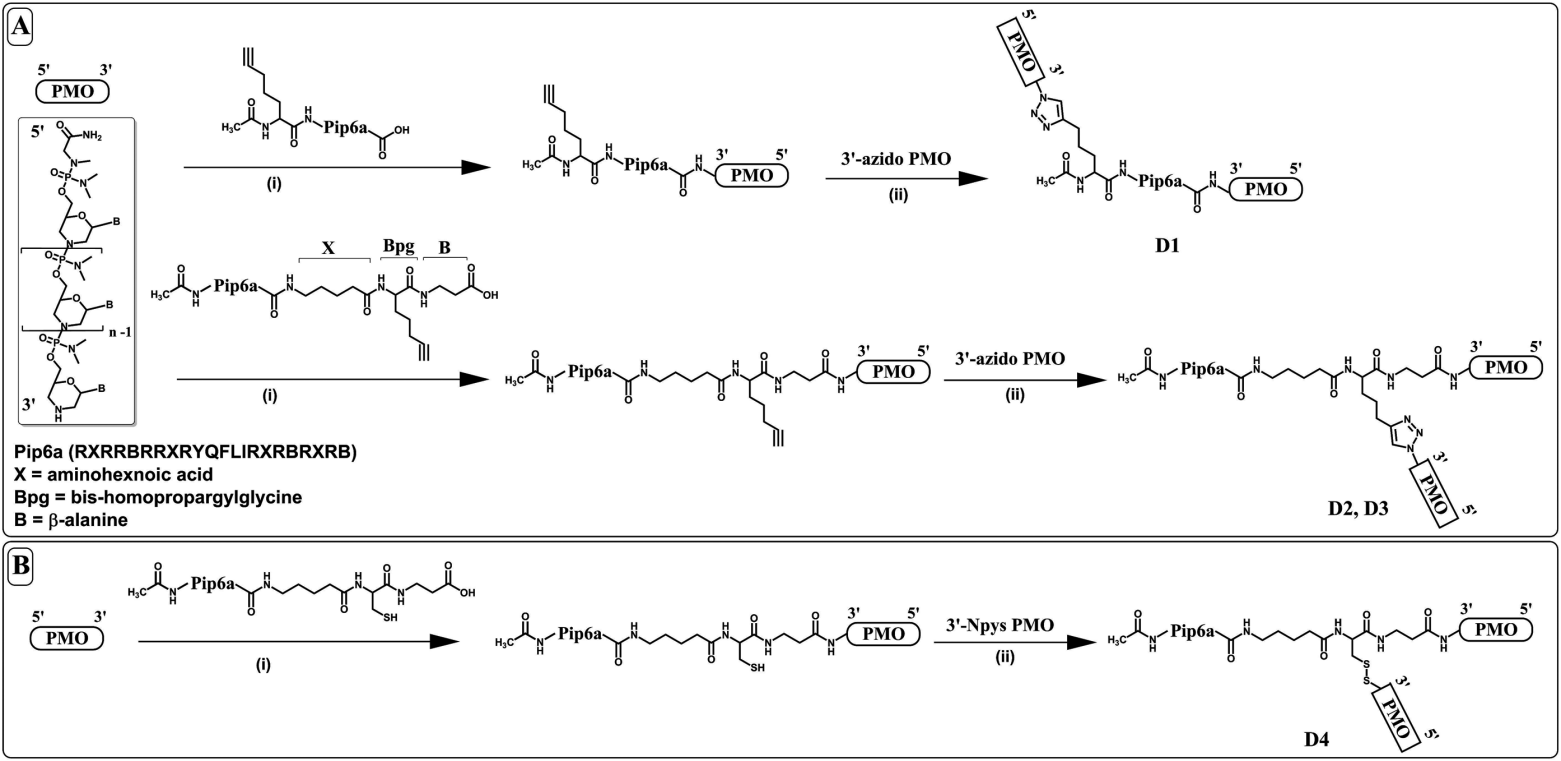
(A and B) (i) Conjugation of the first PMO to the C-terminal carboxylic acid of Pip6a peptide is effected through an amide bond. (**A**) (ii) Alkyne-modified Pip6a-PMO is conjugated through alkyne-azide click chemistry to the azido second PMO using copper (I) to give conjugate D1 (one PMO at each terminus of Pip6a) or D2 and D3 (both PMOs at the C-terminus of Pip6a). (**B**) (ii) Cysteine-modified Pip6a-PMO is conjugated through disulphide bond formation to the thiol-activated second PMO to give conjugate D4.

See Figures 1 and 2 for nomenclature.

### Peptide synthesis

Peptides were synthesized by standard Fmoc chemistry (34) using a CEM Liberty™ microwave peptide synthesizer (Buckingham, UK). Peptides were assembled on Fmoc-β-Ala-OH preloaded Wang resin on a 0.1 mmol scale with excess of Fmoc-protected amino acids, PyBOP and DIPEA (5:5:10). The Nα-Fmoc protecting groups were removed by treating the resin with piperidine in DMF (20% v/v) at 75°C twice, once for 30 s and then for 3 min. The coupling reactions were carried out at 75°C for 5 min. In order to prevent racemization, Fmoc-cysteine (Trt)-OH was coupled at 50°C for 10 min at 60 W microwave power. All amino acids were single coupled except for the arginines, which were double coupled. The Fmoc-l-bis-homopropargylglycine-OH was coupled manually using a 2-fold excess and the coupling success was checked using a TNBS test (35). After completion of peptide assembly, the resin bound peptide was cleaved off by treating the resin with a cocktail of TFA:DoDt:H_2_O:TIPS (94:2.5:2.5:1, v/v/v/v) for 2 h. The peptide was precipitated by addition of ice-cold diethyl ether and washed three times. The crude peptides were analysed and purified to >95% by reversed-phase HPLC (RP-HPLC). The peptide mass characterization was carried out using a MALDI-TOF mass spectrometry (ABI Voyager DE Pro) and an α-cyano-4-hydroxycinnamic acid matrix made up in 70% acetonitrile containing 0.05% TFA.

### Functionalization of PMO

The PMO sequence for exon 23 skipping of *Dmd* pre-mRNA (5′-GGCCAAACCTCGGCTTACCTGAAAT) was either unmodified (standard morpholino with a secondary amine at the 3′ end) or functionalized with a disulfide at its 3′-end. The PMO targeting exon-5 of *Acvr2b* was unmodified (5′-GCCTCGTTTCTCGGCAGCAATGAAC-3′). All PMOs were purchased from Gene Tools LLC (Philomath, USA). 3′-Unmodified PMO was functionalized with an azido group by coupling the free 3′-secondary amine group with Fmoc-azido-l-lysine-OH (Figure 1A). The coupling was carried out by activating the carboxyl group of the amino acid derivative using HBTU (2.5 eq.) and HOAt (2 eq.) in NMP in the presence of 2.5 eq. of DIEA before addition of the PMO dissolved in dimethylsulfoxide (DMSO). The Fmoc-azido-l-lysine–PMO conjugate was then purified using RP-HPLC followed by Fmoc deprotection and purification. In the case of PMO with a disulfide bond at its 3′ end (Figure 1B), the disulfide bond was reduced to give a free sulfhydryl group using a 10-fold excess of tris (2-carboxyethyl)phosphine hydrochloride (TCEP·HCl) in water for 1 h followed by filtration to remove the excess TCEP. The PMO with a free sulfhydryl group was then activated using a 2.5-fold molar excess of 2,2′-dithiobis (5-nitropyridine) (DTNP) in DMSO: acetonitrile (0.1% TFA):H_2_O (0.1% TFA) with (1:1:3) ratios (36). The reaction mixture was stirred at room temperature for 2 h and the NPys-activated PMO was purified by RP-HPLC.

### Peptide–PMO conjugations

Conjugations of peptides to PMOs (Figure 2) were carried out in solution using a 2.5-fold excess of peptide using similar conditions to the coupling of Fmoc-azido-l-lysine-OH to PMO. The conjugation of the second PMO was carried out using either copper (I) mediated alkyne-azide click chemistry between the alkyne-functionalized P-PMO (Figure 2A) and the azide-functionalized second PMO or by forming a disulfide bond between the 3′ NPys-activated second PMO and a free cysteine thiol of the P-PMO (Figure 2B). The alkyne-azide click reaction between azide-functionalized PMO and alkyne functionalized P-PMO was carried out by dissolving the P-PMO in water followed by addition of azido-functionalized PMO (1.2 eq.). Sodium ascorbate (10 eq. as a 20-mM solution) was added and the reaction mixture was vortexed thoroughly followed by addition of copper (II)-TBTA (12 eq. as a 20 mM solution) (37). The click reaction was carried out at room temperature for 6h or at 40°C for 30 min. The conjugation of 3′ NPys-activated PMO to P-PMO was carried out by first dissolving the Npys-activated PMO in ammonium bicarbonate solution (pH 8) followed by addition of the P-PMO dissolved in water. The reaction mixture was stirred at room temperature for 1 h.

The single and dual P-PMO conjugates were purified on a high-resolution (HR)-16 cation-exchange column (GE Healthcare, USA) using 25 mM sodium phosphate buffer (pH 7.2) containing 25% acetonitrile. The conjugates were eluted using a 1 M NaCl solution in the same buffer at a flow rate of 6 ml min^−1^. The excess salts were removed by centrifugation using an Amicon^®^ Ultra-15 3K centrifugal filter device. The conjugates were characterized using MALDI-TOF MS as mentioned above. They were dissolved in sterile water and filtered through a 0.22 μm cellulose acetate membrane (Costar) before use.

### Cell culture and transfection

Mouse H2K/mdx myoblasts were plated at a density of 5 × 10^5^ cells per well in a gelatin (0.01%) pre-coated 24-well plate. H2K/*mdx* myoblasts were grown in high-glucose Dulbecco's modified Eagle's medium (DMEM) supplemented with 20% foetal calf serum (FCS), 2% CEE and 0.002% of interferon-γ at 33°C. The myoblast cells were differentiated into myotubes for the exon-skipping assay. Myoblasts were differentiated for 4 days in DMEM supplemented with 5% HS at 37°C prior to transfection of P-PMOs in serum-free Opti-MEM for 4 h at 37°C. The transfection medium was then replaced with DMEM/5% HS and cells incubated for a further 20 h at 37°C.

### Intramuscular administration

Experiments were carried out in the Biomedical Sciences Unit, University of Oxford according to procedures authorized by the UK Home Office. Experiments were carried out in *mdx* mice (C57BL/10ScSn-Dmd^mdx^/J) (38). Intramuscular (IM) injections (*n* = 3 per treatment) were carried out on 24-week-old *mdx* mice under general anaesthesia. 0.5 nmol peptide–PMO in 30 μl 0.9% saline volume was injected into *tibialis anterior* (TA) muscle. Two weeks post-administration animals were sacrificed by rising CO_2_ inhalation and tissues snap-frozen in a dry ice cooled isopentane bath and stored at −80°C.

### RT-PCR and qPCR analysis

RNA was extracted from either H2K*mdx* cell pellets or from TA tissue sections by mechanical disruption; and subsequently processed using Trizol according to manufacturer's instructions (Life Technologies). RT-PCR analysis of exon skipping levels was carried out with 400 ng of total RNA used as a template in a 50 μl RT-PCR using the GeneAmp RNA PCR kit (Applied Biosystems, Warrington, UK). RT-PCR amplification of the dystrophin *Dmd* transcript was carried out under the following conditions: 95°C for 20 s, 58°C for 60 s and 72°C for 120 s for 30 cycles using the following primers: DysEx20Fo (5′-CAGAATTCTGCCAATTGCTGAG) and DysEx26Ro (5′-TTCTTCAGCTTGTGTCATCC). Two microlitres of this reaction was used as a template for nested amplification using Amplitaq Gold (Applied Biosystems, Warrington UK) under the following conditions: 95°C for 20 s, 60°C for 4 s and 72°C for 120 s for 22 cycles using the following primers: DysEx20Fi (5′-CCCAGTCTACCACCCTATCAGAGC) and DysEx26Ri (5′-CCTGCCTTTAAGGCTTCCTT). *Acvr2b* RT-PCR amplification was carried out under the following conditions: 95°C for 20 s, 60°C for 45 s and 72°C for 60 s for 35 cycles using the following primers: Acvr2bEx4F (5′-CTGCGTTTGGAAAGCTCAGCTCAT) and Acvr2bEx9R (5′-AAGGGCAGCATGTACTCATCGACA). PCR products were analysed on 2% agarose gels. For quantitative analysis of exon skipping levels, 1 μg of RNA was reverse transcribed using the High Capacity cDNA RT Kit (Applied Biosystems, Warrington, UK) according to manufacturer's instructions. qPCR analysis was carried out using 25 ng cDNA template and amplified with Taqman Gene Expression Master Mix (Applied Biosystems, Warrington, UK) on a StepOne Plus Thermocycler (Applied Biosystems, Warrington, UK). Levels of *Dmd* exon 23 skipping were determined by multiplex qPCR of FAM-labelled primers spanning Exons 20–21 (Assay Mm,.PT.47.9564450, Integrated DNA Technologies, Leuven, Belgium) and HEX-labelled primers spanning Exons 23–24 (Mm.PT.47.7668824, Integrated DNA Technologies, Leuven, Belgium). The percentage of *Dmd* transcripts skipping exon 23 was determined by normalizing *Dmd* exons 23–24 amplification levels to *Dmd* exons 20–21 levels. Levels of *Acvr2b* exon 5 skipping were determined by qPCR using FAM-labelled primers spanning exons 5–6 (Mm.PT.58.32079450.g, Integrated DNA Technologies, Leuven, Belgium) and normalized to a cyclophilin B housekeeping gene (Applied Biosystems, Warrington, UK).

### MTS cytotoxicity assay

The levels of cytotoxicity of P-PMOs were assessed in human hepatocytes (Huh7) cells by incubating the cells with P-PMOs at 20 μM for dual Pip6a-PMO (*Dmd*)–PMO (*Acvr2b*) and combination treatment of Pip6a-PMO (*Dmd*) (10 μM) and Pip6a-PMO (*Acvr2b*)) (10 μM). The Huh7 cells were grown to >90% confluency in DMEM/10% FCS in a 96-well plate. The P-PMOs were made up in Opti-MEM without serum and the cells treated for 4 h at 37°C. The Opti-MEM was removed and 100 μl of fresh DMEM/10% FCS was added and the cells were incubated for 20 h at 37°C, followed by addition of 20 μl of CellTiter 96^®^ AQueous One Solution Reagent (Promega, Southampton, UK). The level of cell viability was determined in each case by measuring the absorbance at 490 nm.

## RESULTS

### Bi-specific P-PMO design and synthesis

Novel bi-specific PMO compounds were developed that involved use of standard peptide synthesis methods for synthesis of the functionalized peptide component. The PMO components were obtained using initially unmodified PMO that was then functionalized at its 3′-end with an azido group (Figure 1A) to enable ‘click’ conjugation. Alternatively, a 3′-disulfide functionalized PMO was used to prepare a 3′-NPys-activated PMO (Figure 1B). The yield of 3′-Npys PMO was 55%, whereas the 3′-azido PMO was in lower yield of 19.5% because of the two-step RP-HPLC purification used.

The CPP chosen for the constructions was Pip6a (Figure 2A). Two different types of bi-specific compounds were designed. In the first, the two PMO oligonucleotides were each conjugated to a different end of the Pip6a peptide (Figure 2A) (designated D1) or in the second where the PMO oligonucleotides were both attached at the carboxy-terminal end of Pip6a (designated D2 and D3). In the case of click chemistry conjugation to PMO, Pip6a was synthesized with an alkyne group either at its N-terminus for D1, or at its C-terminus for D2 and D3 (Figure 2A). In D1, PMO (*Dmd*) was conjugated to the C-terminus through an amide bond and the 3′-azido-PMO (*Acvr2b*) was conjugated at the N-terminus through a triazole bond. For bi-specific conjugate D2, the *Acvr2b* PMO was click conjugated, whereas in conjugate D3 the *Dmd* targeting PMO was click conjugated. For click conjugations at the C-terminus of Pip6a, one β-alanine (B) and one aminohexanoic acid (X) spacer residue were incorporated on either side of the Bpg alkyne derivative, whereas no spacer was used for N-terminal click conjugations (Figure 2A). Bi-specific conjugate D4 was prepared with a similar spacing to D3, but where a disulfide bond replaced the triazole bond through synthesis of Pip6a having a C-terminal X-Cys-B extension (Figure 2B). The assembly of these bi-specific conjugates required synthesis of three different derivatives of Pip6a, which were synthesized on solid phase using Fmoc peptide chemistry and purified by RP-HPLC to >95% purity in yields of 50–60%.

**Figure 3.**
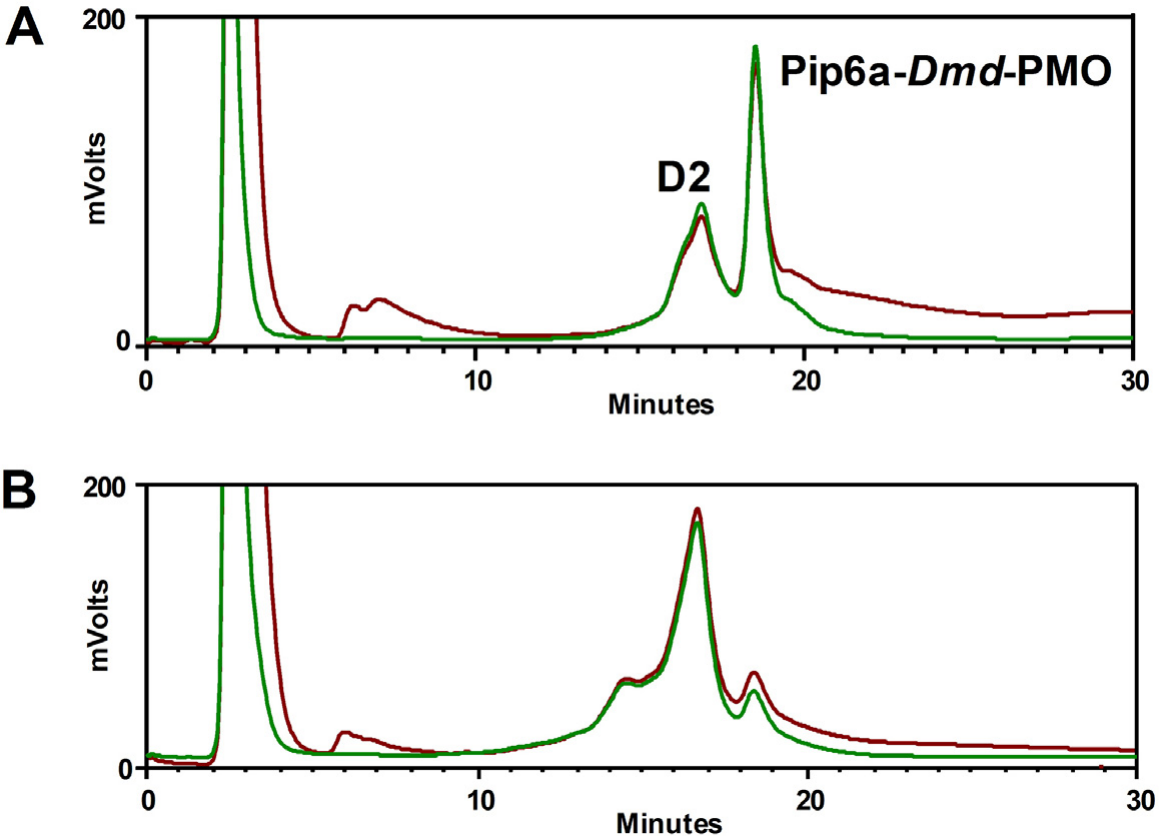
Cation-exchange chromatogram in the purification of bi-specific conjugate D2 synthesized by alkyne-azide click chemistry. The copper-(I)-mediated click reaction was carried out using similar conditions but in (**A**) at room temperature for 6 h and in (**B**) at 40°C for 30 min.

For each construct, conjugation of the first PMO to each of the three Pip6a derivatives was carried out through amide bond formation between the C-terminal carboxylic acid group of the peptide to the secondary amine at the 3′-end of the PMO, similarly to the synthesis of Pip6a-PMO (*Dmd*) (18) (Figure 2A and B). The conjugations were carried out in solution and purifications were carried out by ion exchange HPLC. Isolated yields of P-PMO were 35–40% (based on the amount of starting PMO). The 3′-azido PMO was coupled to the alkyne-P-PMO using copper (I)-mediated alkyne-azide click chemistry resulting in a yield of 42% for conjugate D1 and 38% for conjugates D2 and D3 (Figure 2A). The click reaction for syntheses of D2 and D3 was sluggish at room temperature and after 6 h only a small amount of bi-specific conjugate was formed (Figure 3A). However, heating the reaction mixture to 40°C significantly improved the reaction rate and after 30 min the reaction had proceeded to near completion as determined by ion exchange HPLC (Figure 3B). The 3′-Npys PMO (*Dmd*) was conjugated to P-PMO (*Acvr2b*) to give a disulfide bond in bi-specific conjugate D4 (Figure 2B) in a yield of 58%. Mass spectral characterizations of conjugates D1 to D4 and their intermediates are shown in Table 1.

### Evaluation of dystrophin and activin exon-skipping activities of bi-specific P-PMOs

**Figure 4.**
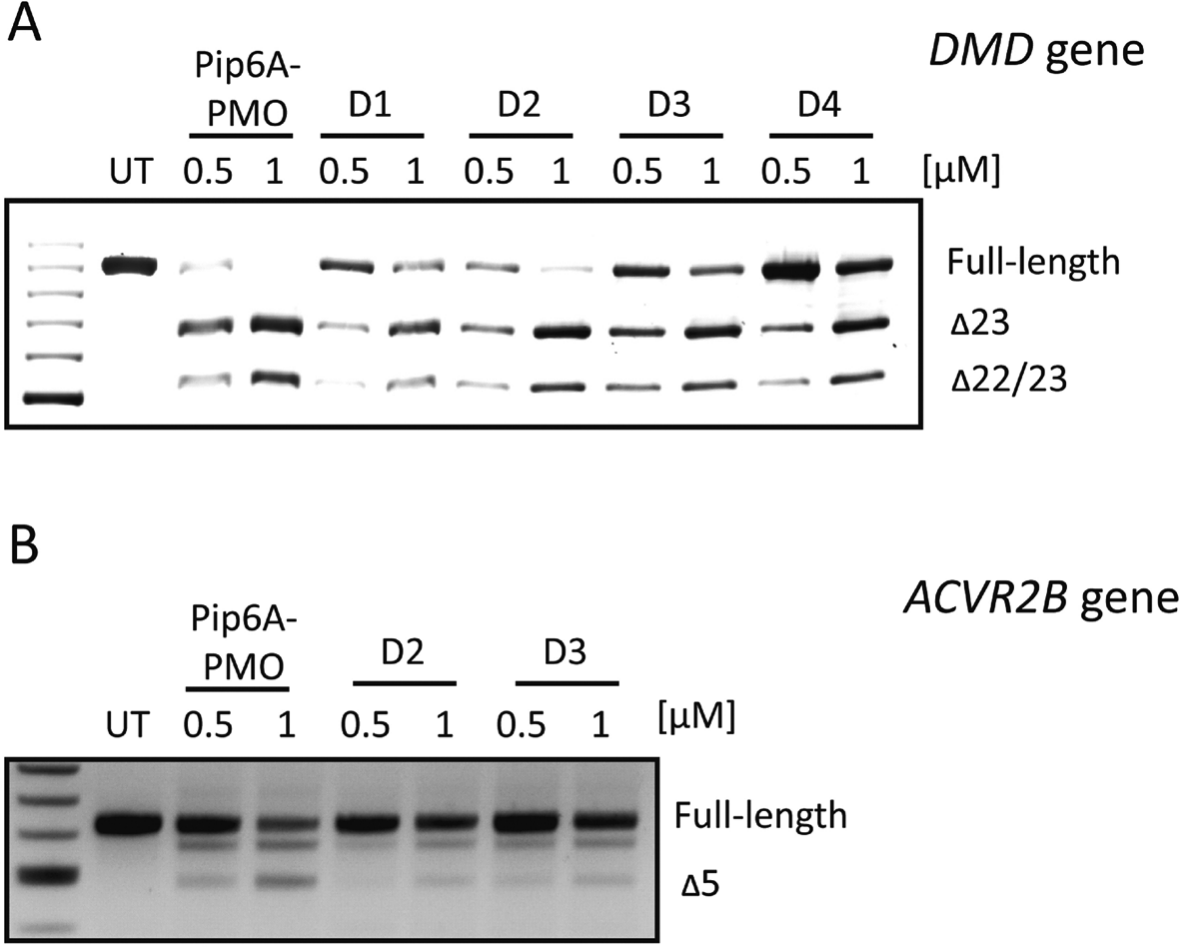
RT-PCR analysis of (**A**) *Dmd* and (**B**) *Acvr2b* exon skipping activity of singular and bi-specific P-PMO conjugates in *H_2_K mdx* cells treated for 4 h with 0.5 or 1 μM concentrations of conjugates.

The efficacies of the bi-specific P-PMO constructs were assessed in an initial screening step by RT-PCR using *Dmd* exon 23 skipping in H2K *mdx* cells (Figure 4A). For all conjugates tested, high levels of exon 23 skipping were found in a dose-dependent manner (with some exons 22–23 double skipping which is frequently observed in this test system). The bi-specific conjugates D2 and D3, where both PMOs are conjugated to the C-terminus of Pip6a, exhibited better exon-skipping activity than for D1 and D4. Since bi-specific conjugate D4 was less effective than D3, this suggests that there is no advantage to use of a cleavable disulfide linkage over use of stable click chemistry for addition of the second PMO. The control singly conjugated Pip6a-PMO (*Dmd*) demonstrated only slightly higher exon skipping compared to conjugates D2 and D3.

Bi-specific conjugates D2 and D3, which showed the best *Dmd* exon skipping levels, were examined further for their exon-skipping efficacies in *Acvr2b* (Figure 4B). The results for *Acvr2b* mirrored that of *Dmd* targeting. Only very slightly higher exon skipping was observed with the control singly conjugated Pip6a-PMO compared to bi-specific counterparts D2 and D3. In addition, the general level of exon 5 skipping in the *Acvr2b* gene was found to be lower than that for *Dmd*. This is potentially because disruptive exon skipping by oligonucleotides for this gene is harder to achieve than skipping of *Dmd* exon 23 and has not been fully optimized as yet. Bi-specific conjugate D3 was marginally more efficient than for D2.

### Evaluation of dystrophin and activin exon-skipping activity in *mdx* mice

**Figure 5.**
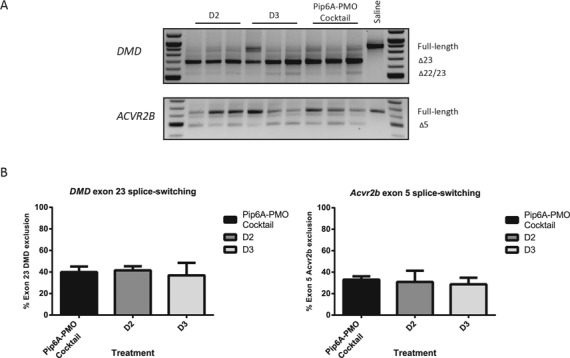
Splice-switching activities of bi-specific (D2, D3) and a 1:1 molar cocktail of singly conjugated Pip6a-PMOs following intramuscular injection (*n* = 3) into the *tibialis anterior* muscle of *mdx* mice. (**A**) RT-PCR analysis of *Dmd* exon 23 and *Acvr2b* exon 5 removal from the mature transcripts. (**B)** Quantitative PCR analysis of *Dmd* exon 23 and *Acvr2b* exon 5 skipping activity. Error bars = S.E.M.

Since both D2 and D3 conjugates demonstrated skipping activity of both genes in cells and had the highest levels of *Dmd* exon 23 skipping, they were further evaluated *in vivo* in the *mdx* mouse model of DMD. Intramuscular administration of 0.5 nmol of either the D2, D3 bi-specific conjugates was carried out into the TA muscle of *mdx* mice and compared with a 1:1 molar cocktail of singly conjugated Pip6a-PMOs. Analysis of splice-switching activity was carried out 2 weeks post-administration. Each of the CPP–PMO conjugates demonstrated robust splice-switching activity, with higher splice-switching activity evident for *Dmd* targeting compared to *Acvr2b*, as was also seen in the *in vitro* cell culture studies. Since no significant differences between the constructs could be seen following gel analysis by RT-PCR in the case of *Dmd* gene targeting (Figure 5A), quantitative analysis of splice switching was carried out using qPCR primers to determine the reduction in the level of transcripts containing the target exons. In each case 35–40% of exon 23 skipped *Dmd* transcripts were found, when normalized to *Dmd* transcripts from non-injected control muscle, with no statistically significant differences seen between P-PMO treated mice for both singly conjugated and bi-specific conjugates. Unsurprisingly, the pattern of exon skipping was also maintained for *Acvr2b* gene targeting, where there were no significant differences seen between D2 or D3 conjugates or the singly conjugated Pip6a-PMO counterpart (Figure 5B).

### Cell viability

**Figure 6.**
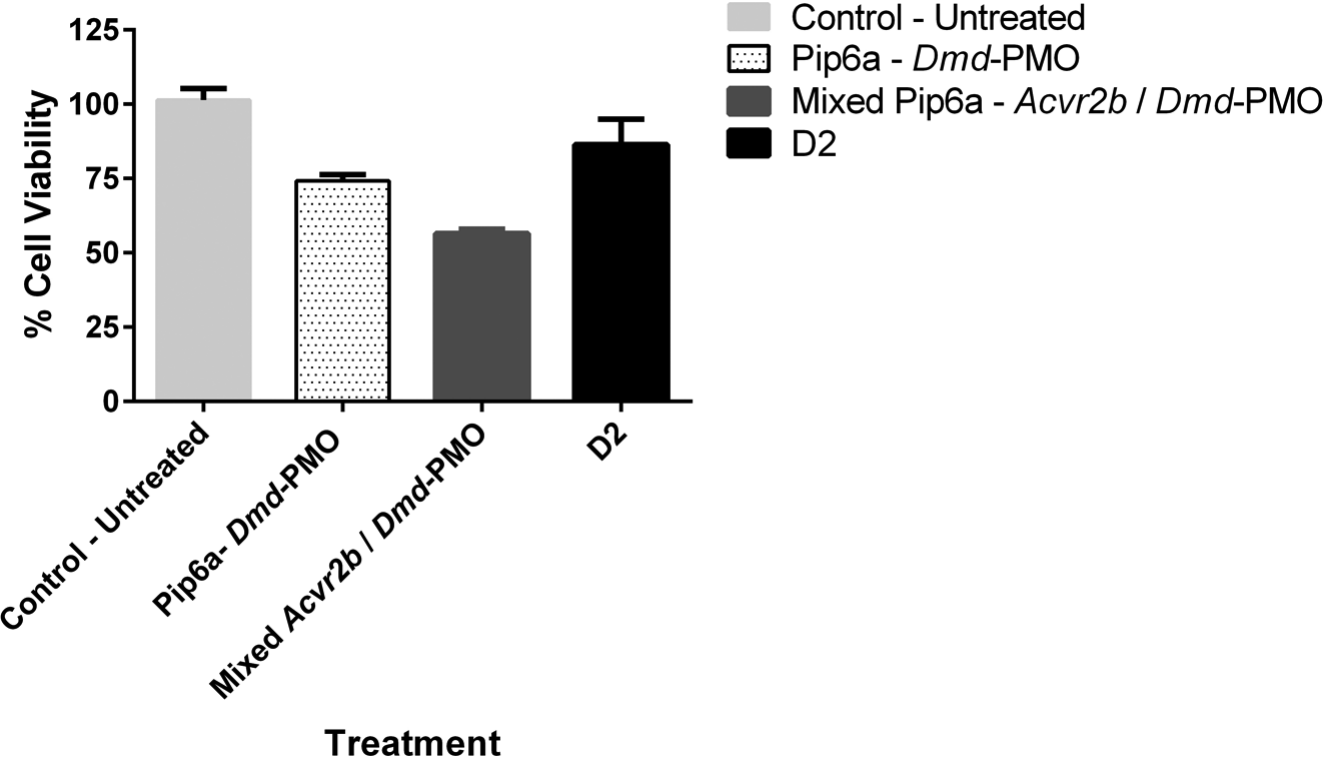
Evaluation of the cell viabilities of Pip6a-PMO (*Dmd*) (20 μM), a mixture of Pip6a-PMO (*Dmd*) and Pip6a-PMO (*Acvr2b*) (10 μM each), and bi-specific P-PMO conjugate D2 (20 μM) in human hepatocytes (Huh7).

Cell and *in vivo* toxicities of P-PMOs are known to be predominantly a function of the peptide component and are dose-dependent (39). Therefore, the cell viability of the D2 bi-specific conjugate was assessed in human hepatocytes (Huh7) cells and compared to that of Pip6a-PMO (*Dmd*) and a mixture of the two Pip6a-PMOs against the two different targets *Dmd* and *Acvr2b*, in each case using a high equimolar concentration based on total PMO. Thus 20 μM of bi-specific conjugate D2 was compared with a mixture of 10 μM each of Pip6a-PMO (*Dmd*) and Pip6a-PMO (*Acvr2b*) and the percentage of cell survival measured (Figure 6). These results showed significantly higher cell viability for the bi-specific D2 conjugate compared to a mixture of both Pip6a-PMOs for the two individual targets.

## DISCUSSION

The promise of SSOs as therapeutic agents is being realized, with a number of clinical trials for DMD in progress to assess the efficacy of targeting a single exon (exon 51) to by-pass disease causing mutations (8–10). Further clinical trials are also being undertaken to target other exons, notably in the region covering exons 45–55 (40). Multiple simultaneous exon skipping using a cocktail of SSOs has been suggested as an approach to target a majority of patients (40,41). A proof-of-concept for multiple exon skipping was demonstrated in the golden retriever model of DMD (42) as well as in human patient cells lines (43). The concept was further extended to the targeting of exons 45–55 using cocktails of various SSOs (44). Unsurprisingly, the levels of exon skipping found to date have been low. In an attempt to improve this efficacy, studies were undertaken in the *mdx*52 mouse model using a cocktail of 10 different ‘vivo-morpholino’ PMOs to delete the entire stretch of exons 45–55 (45). Although a skipped transcript could be detected in these mice, the significant likely toxicity of delivering into mice 10 individual PMOs that are functionalized with guanidinium groups might hinder their clinical development.

Instead of the use of a cocktail of P-PMOs and with the need in mind to minimize toxicity, we sought to develop proof-of-concept for use of bi-specific PMO SSOs that could simultaneously target two different exons in different genes rather than in the same gene. We chose for these initial studies simultaneous targeting of exon 23 of the *Dmd* gene and exon 5 of the *Acvr2b* gene, using a single CPP as PMO delivery agent and to observe whether exon skipping could be maintained for both targets. Dumonceaux *et al.* showed that a combination of restoration of dystrophin and simultaneously inhibition of the myostatin-signalling pathway results in a significant improvement in muscle growth and force in dystrophic *mdx* mice (25). In a similar study, myostatin knockdown in conjunction with dystrophin restoration using an exon-skipping approach using a cocktail of two separate Pip6a-PMOs resulted in significantly increased mouse muscle mass (32). Thus targeting two genes in this way might be expected to have clinical relevance.

We used our well-characterized Arg-rich CPP Pip6a as the model CPP because of the known high level of exon skipping observed for PMO conjugates in the DMD model (18) and we designed orthogonal conjugation chemistries using amide, disulfide and triazole bonds. The first conjugation of PMO to Pip6a was carried out in all cases through formation of a stable amide bond between the 3′-secondary amine of the PMO to the C-terminal carboxyl group of a synthetic Pip6a derivative. The second PMO conjugation was then effected either using an alkyne group on the Pip6a to an azide-functionalized PMO to give a stable triazole linkage or with a Cys residue on the Pip6a to an activated thiol group on the PMO to form a reversible disulfide linkage (Figures 1 and 2). Bi-specific conjugate D1 has one PMO at each Pip6a terminus whilst D2 and D3 have both PMOs at the C-terminus of Pip6a. The more sluggish click conjugation of the second PMO to the C-terminus of Pip6a in D2 and D3 at room temperature was presumably due to poorer accessibility than in the case of D1, but heating to 40°C readily facilitated triazole bond formation (Figure 3). Conjugation of the second PMO using a disulfide bond (D4) did not require heating.

In *mdx* muscle cells, the lowest exon-skipping efficacy was observed for bi-specific conjugate D1 that had a PMO conjugated to each end of the Pip6a, which suggests that the ability of Pip6a to deliver PMO through the endosomal pathway and into the nucleus is inhibited by placing a bulky PMO at its N-terminus. By contrast, when both PMOs were placed at the C-terminus of the Pip6a (D2 and D3), the *Dmd* skipping activity was restored to close to the level observed with Pip6a-PMO (*Dmd*) (Figure 4A), which confirms that CPP delivery is optimal when the CPP N-terminus is not blocked by a bulky substituent. Both conjugates D2 and D3 also demonstrated high levels of *Acvr2b* exon skipping in cells and close to that of the single Pip6a-PMO (*Acvr2b*) (Figure 4B). Further, both D2 and D3 conjugates showed exon skipping of both the Dmd and Acvr2b targets by intramuscular delivery (Figure 5) at levels unaltered from that of individual Pip6a-PMOs. These results show conclusively that there is no sequestration of one PMO at its own target that prevents the action of the other PMO at its own target. Thus, there must be a sufficient on-off equilibrium established for each target release and for target accessibility. This is an important finding that validates the use of bi-specific P-PMO.

Interestingly bi-specific P-PMO D4, in which the PMO (*Dmd*) was conjugated through a reducible disulfide bond, was less active than stably conjugated D2 and D3 constructs (Figure 4). One explanation for this could be due to partial reduction of the disulfide bond upon cell entry and liberation of free *Dmd* PMO from the bi-specific conjugate before the Pip6a can deliver it into the cell nucleus. However, note that no difference was found in splicing redirection in a HeLa705 cell model using either disulfide-linked or stably linked R_6_-Penetratin PNA (46). By contrast in a recent study, bi-specific 2′-*O*-methyl phosphorothioate SSOs targeting both the *Mstn* and *Dmd* genes and delivered using a cationic transfection reagent were more effective when linked through a cleavable disulfide linker than through a non-cleavable hydrocarbon linker (33). The differential effect of use of a cleavable disulfide linkage probably reflects a different cell and nuclear uptake mechanism for SSOs delivered by a transfection agent compared to covalent CPP delivery of uncharged PMO or PNA. Detailed mechanistic studies would be needed to confirm this, but it should be noted that uptake of Pip6a-PMO (*Dmd*) into *mdx* skeletal muscle cells has been shown recently to be predominantly caveolae-mediated, whereas in cardiomyocytes uptake was mostly clathrin-mediated (47), suggesting that uptake routes are also cell dependent in addition to being dependent on oligonucleotide type and delivery method.

For the future it should be noted that the efficiency of exon 5 skipping for PMO (*Acvr2b*) is not as high as for exon 23 skipping for PMO (*Dmd*). PMO (*Acvr2b*) will therefore need further optimization to improve efficiency on this target. Alternatively, one might prefer to use a combination of PMO (*Dmd*) and a PMO that targets myostatin directly (32) to make a bi-specific PMO rather than targeting its receptor, and thus this second PMO needs to be optimized before embarking on lengthy intravenous delivery studies in *mdx* mice where larger scale synthesis would be needed. Clearly such studies need to investigate physiology benefits in addition to exon skipping (32). However, the increase in cell viability in human hepatocyte cultures for bi-specific conjugate D2 compared to the same PMO concentration for a mixture of two Pip6a-PMOs (Figure 6) is encouraging and if this lower toxicity is maintained in systemic delivery, the clinical importance could be significant when considering multiple targeting (whether targeting different exons in the same gene or in two different genes).

In summary, we have developed new synthetic methodology for conjugation of two PMO SSOs to a single Pip6a CPP and shown efficient targeting in cells and *in vivo* of two separate genes with retained potency for each SSO. This work should enable further systemic studies on multiple SSO targeting in both DMD and also potentially in other neuromuscular disease models.
